# Potential novel therapeutic strategies for neuropathic pain

**DOI:** 10.3389/fnmol.2023.1138798

**Published:** 2023-04-21

**Authors:** Zelu Du, Jian Zhang, Xu Han, Weifeng Yu, Xiyao Gu

**Affiliations:** Department of Anesthesiology, Renji Hospital, Shanghai Jiao Tong University School of Medicine, Shanghai, China

**Keywords:** neuropathic pain, clinical trial, drug targets, physical means, therapeutic strategies

## Abstract

**Purpose:**

To explore the potential therapeutic strategies of different types of neuropathic pain (NP) and to summarize the cutting-edge novel approaches for NP treatment based on the clinical trials registered on ClinicalTrials.gov.

**Methods:**

The relevant clinical trials were searched using ClinicalTrials.gov Dec 08, 2022. NP is defined as a painful condition caused by neurological lesions or diseases. All data were obtained and reviewed by the investigators to confirm whether they were related to the current topic.

**Results:**

A total of 914 trials were included in this study. They were divided into painful diabetic neuropathy (PDN), postherpetic neuralgia (PHN), sciatica (SC), peripheral nerve injury-related NP (PNI), trigeminal neuralgia (TN), chemotherapy-induced NP (CINP), general peripheral NP (GPNP) and spinal cord injury NP (SCI-NP). Potential novel therapeutic strategies, such as novel drug targets and physical means, were discussed for each type of NP.

**Conclusion:**

NP treatment is mainly dominated by drug therapy, and physical means have become increasingly popular. It is worth noting that novel drug targets, new implications of conventional medicine, and novel physical means can serve as promising strategies for the treatment of NP. However, more attention needs to be paid to the challenges of translating research findings into clinical practice.

## 1. Introduction

Neuropathic pain (NP) is a significant contributor to the global burden of disease. Its prevalence varies between 6.9 and 10% in the general population, and is expected to increase by 2–3% annually, as the older population grows and the incidence of diabetes and cancer increases (Van Hecke et al., 2014). NP leads to a substantial economic burden on healthcare resources and societal costs. It is estimated that the direct medical costs of NP would exceed 635 billion per year in the United States (Gaskin and Richard, 2012). Most patients with NP complain of persistent or intermittent spontaneous pain (Finnerup et al., 2021). Lesions or diseases involving the somatosensory nervous system not only lead to a loss of function, but also increase pain sensitivity and spontaneous pain (Scholz et al., 2019). Moreover, NP patients are usually associated with severe anxiety, depression, sleep disturbances, and even suicide. However, the treatment of NP remains unsatisfied and challenging.

The current recommendations for the pharmacological treatment of NP were published by NP Special Interest group (NeuPSIG) of the International Association for the Study of Pain (IASP). Drugs with moderate-to-high quality of evidence and strong recommendation include tricyclic antidepressants (TCA), gabapentin, pregabalin, and serotonin noradrenaline reuptake inhibitors (SNRIs; duloxetine and venlafaxine), which are recommended as first-line therapies. The less recommended drugs include capsaicin 8% patches and lidocaine patches (Finnerup et al., 2015). Opioids and subcutaneous injections of botulinum-toxin type A for peripheral NP are weakly graded and recommended for third-line therapies (Finnerup et al., 2021). Opioids should be reserved for patients who do not respond to other treatment options and are at low risk of adverse reactions. Besides, the efficacy of analgesics (e.g., NSAIDs) in NP patients is still unclear (Scholz et al., 2019).

Although the guidelines recommend multiple therapeutic interventions, the first-line drugs provide unsatisfactory pain relief in the clinic (Finnerup et al., 2015). Gabapentin and pregabalin, which target voltage-dependent calcium channels α2δ1 subunit (Li et al., 2019), perform well for postherpetic neuralgia (PHN) and painful diabetic neuropathy, but not for sciatica and migraine (Finnerup et al., 2015). The common side effects of gabapentin and pregabalin include dizziness, drowsiness, euphoria, peripheral edema and weight gain (Finnerup et al., 2021). TCA and SNRIs inhibit presynaptic reuptake of serotonin and norepinephrine (Kremer et al., 2016). The common side effects of TCA include dry mouth, drowsiness and cardiovascular related effects, while SNRIs have side effects of nausea, insomnia and hyperhidrosis at an early stage (Kremer et al., 2016; Attal and Bouhassira, 2021). Lidocaine acts through a dependent voltage-gated blockade of sodium channels (Devor et al., 1992). The absorption of lidocaine into plasma leads to a potential risk of systemic adverse reactions. Capsaicin binds to TRPV1, and repeated or single application of high concentrations can cause reversible effects of intraepidermal nerve fiber density and pain receptor desensitization (Anand and Bley, 2011). The application of a capsaicin patch can lead to local skin redness or itching (Backonja et al., 2008). Hence, it is of great importance to develop novel therapeutic strategies for the clinical treatment of NP.

To achieve this goal, researchers are exploring new ways. With the rapid development of science and technology, scientists and clinicians explored deeper understandings of the mechanism of NP. Besides, several novel therapeutic strategies are invented and tested based on these mechanisms. For example, methadone is now being tested for the clinical management of NP (NCT05235191). Transcutaneous pulsed radiofrequency therapy is applied to test its effectiveness in the alleviation of PDN symptoms (NCT05480527). In this review, we searched the currently ongoing or completed clinical trials on NP treatment through ClinicalTrials.gov, which is the largest resource of the clinical research website of the US National Library of Medicine. This review aimed to explore new therapeutic strategies for different types of NP and to summarize the cutting-edge novel potential approaches for NP treatment.

## 2. Materials and methods

### 2.1. Data collection and processing

ClinicalTrials.gov was used to search the relevant clinical trials. The search was done on December 08, 2022 by ZL Du and J Z. NP is defined as a painful condition caused by neurological lesions or diseases. All trials were downloaded as a.tsv file, and then reviewed by WF Yu and XY Gu to confirm whether they were related to the current topic. Several diseases, such as complex regional pain syndrome type 1, low back pain without radicular pain, fibromyalgia and atypical facial pain, were not included because they did not meet the current definition of NP (Finnerup et al., 2016). The inclusion criteria of key trials were as follows: (1) completed within 2 years; (2) recruiting or not yet recruiting; (3) did not include drugs which were recommended as first-line therapy.

The classification of NP was defined as peripheral neuropathic pain (PNP) or central neuropathic pain (CNP) according to ICD-11 (Table 1). The trials related to NP treatment were further collected and summarized. The intervention was systemic or topical treatment for at least 3 weeks.

**Table 1:** Classification of chronic neuropathic pain in ICD-11.

PNP (peripheral neuropathic pain)	CNP (central neuropathic pain)
Painful polyneuropathy (include Painful diabetic neuropathy and Chemotherapy-induced neuropathic pain)	Contains central neuropathic pain associated with spinal cord injury
Postherpetic neuralgia	Central neuropathic pain associated with brain injury
Painful radiculopathy (include Sciatica)	Central post-stroke pain
Postherpetic neuralgia neuropathic pain after peripheral nerve injury (PNI-NP)	Central neuropathic pain caused by multiple sclerosis
Trigeminal neuralgia (TN)	Other specified and unspecified chronic central neuropathic pain
Other specified and unspecified chronic peripheral neuropathic pain	

## 3. Results

### 3.1. Overview of the included clinical trials

**Figure 1:**
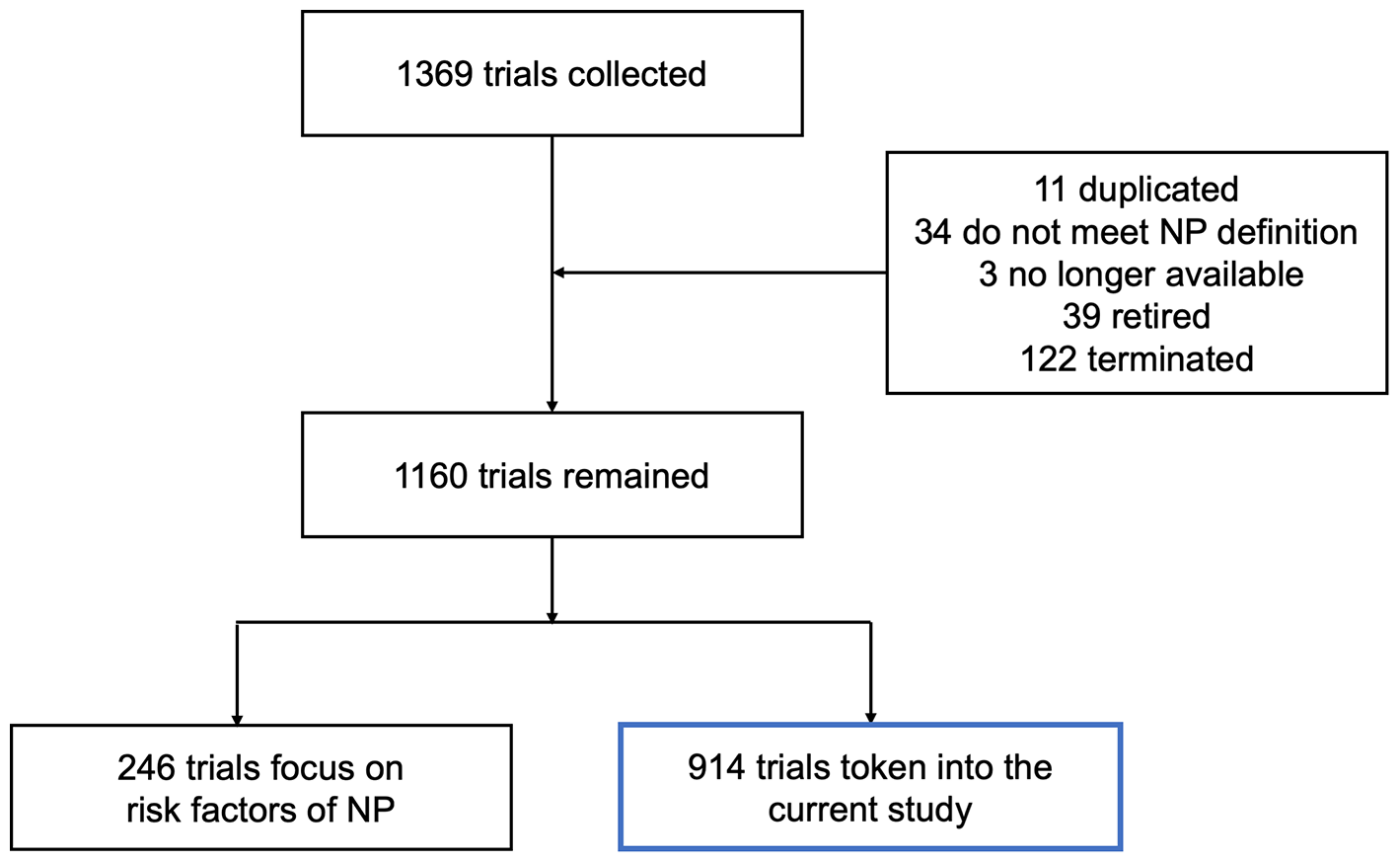
Flow chart of the current study.

**Figure 2:**
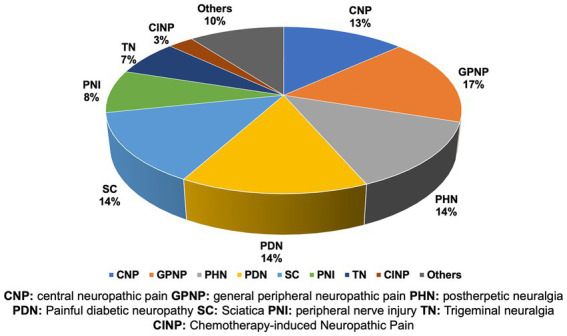
The proportion of different type of neuropathic pain in clinical trials.

A total of 1,369 clinical trials related to NP were identified. After reviewing, there were 11 duplicated trials, 34 trials did not meet the NP definition, 3 were no longer available, 39 were retired, and 122 were terminated. Meanwhile, 246 trials focused on the risk factors, objective measures and economic burden of NP, as well as its effect on other functions. As a result, 914 trials were included in this study (Figure 1). Among them, 793 trials were focused on peripheral NP (PNP), including 132 painful diabetic neuropathy (PDN), 123 postherpetic neuralgia (PHN), 125 sciatica (SC), 76 peripheral nerve injury-related NP (PNI), 64 trigeminal neuralgia (TN), 25 chemotherapy-induced NP (CINP), 154 general PNP (GPNP) and others (including pudendal neuralgia, post-amputation NP, HIV-related NP). The remaining 121 trials were focused on central NP (CPN). Almost half of them (54 of 121) were concentrated on spinal cord injury NP (SCI-NP; Figure 2). The potential therapeutic strategies for NP are summarized as follows.

## 4. Peripheral neuropathic pain

### 4.1. Painful diabetic neuropathy

PDN is a frequent subtype of PNP, which is defined as “pain as a direct consequence of abnormalities in the peripheral somatosensory system of diabetes patients” (Jensen et al., 2011). PDN is one of the painful polyneuropathy. The prevalence of PDN ranged from 13 to 35% in patients with diabetes mellitus (Ponirakis et al., 2021). PDN is a frequent complication of long-term diabetes, and one of the leading causes of morbidity and disability (Rosenberger et al., 2020). Due to the improvement in living standards and increased prevalence of diabetes, PDN has attracted considerable attention, with the second most trials (132/914, 14.4%) until now. Pregabalin and duloxetine, together with tapentadol as a supplement, is the current first-line therapy for PDN according to the FDA recommendation (Rosenberger et al., 2020). Unwanted side effects are the main issue. The ongoing trials for PDN treatment are mainly focused on novel molecular targets and physical means.

#### 4.1.1. Potential drugs

In NCT05219812, a new drug BAY 2395840 that blocks bradykinin receptor B1 (BDKRB1) is being tested (Table 2, Line 1). BDKRB1 was upregulated on sensory C-fibers, astrocytes and microglia in the spinal cord of streptozotocin (STZ)-induced diabetic rats, and peripheral blockade of BDKRB1 reversed tactile and cold allodynia at 3–6 h post-treatment in diabetic rats (Talbot et al., 2010). Inhibiting BDKRB1/RAS/MEK signaling pathway exhibited a better outcome in NP (Xie et al., 2021). The current trial by Bayer aimed to test the effectiveness and safety of BAY2395840 on participants who suffered from PDN.

**Table 2:** Clinical Trials of different type of neuropathic pain.

No.	Type of NP	NCT number	Status	Sample size	Study title	Design
1	PDN	NCT05219812	Active, not recruiting	80	A randomized, double-blind, cross-over, placebo-controlled, multi-center, phase 2a study to assess the safety and signal in efficacy of BAY 2395840 in patients with diabetic neuropathic pain	Allocation: randomized |intervention model: crossover assignment masking: double (participant, investigator) | primary purpose: treatment
2	PDN	NCT03176472	Active, not recruiting	282	A Phase 2, randomized, double-blind, placebo-controlled, parallel group study of ricolinostat in patients with painful diabetic peripheral neuropathy	Allocation: randomized Intervention Model: parallel assignment masking: quadruple primary purpose: treatment
3	PDN	NCT04146896	Completed	229	A randomized, double-blind, placebo-controlled study to assess the efficacy and safety of NYX-2925 in subjects with neuropathic pain associated with diabetic peripheral neuropathy	Allocation: randomized intervention model: parallel assignment | masking: triple (participant, investigator, outcomes assessor) primary purpose: treatment
4	PDN	NCT05322746	Recruiting	102	Effects of focal vibrations on neuropathic pain in diabetic peripheral neuropathy	Allocation: randomized intervention model: parallel assignment | masking: none (open label) | primary purpose: treatment
5	PDN	NCT05469074	Recruiting	80	Optimization of ESStim for the diabetic neuropathic pain treatment Phase II study	Allocation: randomized intervention model: parallel assignment | masking: triple (participant, investigator, outcomes assessor) | primary purpose: treatment
6	PDN	NCT04833660	Not yet recruiting	22	The effect of repetitive transcranial magnetic stimulation on diabetic peripheral neuropathic pain: a randomized controlled trial	Allocation: randomized intervention model: parallel assignment | masking: triple (participant, investigator, outcomes assessor) primary purpose: treatment
7	PDN	NCT05104047	Recruiting	44	Moxibustion for neuropathic pain in type 2 DM	Allocation: randomized intervention model: parallel assignment | masking: triple (participant, investigator, outcomes assessor) primary purpose: treatment
8	PDN	NCT04455633	Completed	319	A Phase 2, randomized, double-blind, placebo-controlled, parallel-group, multicenter study to evaluate the efficacy, safety, and pharmacokinetics of LX9211 in the treatment of diabetic peripheral neuropathic pain	Allocation: randomized intervention model: parallel assignment masking: quadruple (participant, care provider, investigator, outcomes assessor) primary purpose: treatment
9	PHN	NCT04662281	Active, not recruiting	74	A Phase 2, double-blind, randomized, placebo-controlled, parallel-group study to evaluate the efficacy and safety of LX9211 in the treatment of postherpetic neuralgia	Allocation: randomized intervention model: parallel assignment | masking: quadruple (participant, care provider, investigator, outcomes assessor) primary purpose: treatment
10	PHN	NCT04664530	Recruiting	48	The study on the esketamine in the treatment of postherpetic neuralgia	Allocation: randomized intervention model: parallel assignment | masking: quadruple (participant, care provider, investigator, outcomes assessor) primary purpose: treatment
11	PHN	NCT04313335	Recruiting	750	Prophylactic duloxetine administration during acute herpes zoster prevents postherpetic neuralgia	Allocation: randomized intervention model: parallel assignment | masking: single (outcomes assessor) | primary purpose: prevention
12	PHN	NCT04560361	Recruiting	448	The analgesic effect of electroacupuncture on postherpetic neuralgia: a multicenter randomized controlled trial	Allocation: randomized intervention model: parallel assignment | masking: triple (participant, investigator, outcomes assessor) primary purpose: treatment
13	PHN	NCT03584061	Completed	10	Treatment of chronic postherpetic pain with fat grafting–a pilot study	Allocation: N/A | intervention model: single group assignment | masking: none (open label) | primary purpose: treatment
14	PHN	NCT05140863	Recruiting	345	A multicenter, randomized, double-blind, placebo-controlled, 12-week, phase 3 study to evaluate the efficacy and safety of HSK16149 capsules in chinese patients with postherpetic neuralgia	Allocation: randomized intervention model: parallel assignment masking: double (participant, investigator) primary purpose: treatment
15	SC	NCT03347929	Recruiting	150	NSAIDs in sciatica (NIS), an investigator initiated randomized placebo controlled trial of naproxen	Allocation: randomized intervention model: parallel assignment | masking: quadruple (participant, care provider, investigator, outcomes assessor) primary purpose: treatment
16	SC	NCT05000658	Recruiting	106	Efficacy of soluble dexamethasone by echo-guided infiltration through the sacrococcygeal hiatus in refractory sciatica: a prospective randomized double-blind study versus placebo	Allocation: randomized intervention model: parallel assignment | masking: quadruple (participant, care provider, investigator, outcomes assessor) primary purpose: treatment
17	SC	NCT05196503	Recruiting	60	Efficacy of platelet rich fibrin in the prevention of residual neuropathic pain following disk herniation surgery	Allocation: randomized intervention model: parallel assignment | masking: double (participant, outcomes assessor) primary purpose: treatment
18	SC	NCT04875741	Recruiting	72	Comparative effects of neural mobilization and muscle energy technique on pain, range of motion and functional disability in patients with sciatica: a randomized controlled trial	Allocation: randomized intervention model: parallel assignment | masking: double (participant, outcomes assessor) primary purpose: treatment
19	SC	NCT05129540	Not yet recruiting	80	Foot orthoses in patients with chronic sciatica caused by lumbar disk herniation: a randomized clinical trial	Allocation: randomized intervention model: parallel assignment | masking: double (participant, investigator) primary purpose: treatment
20	PNI	NCT04967664	Recruiting	408	An interventional, Phase III, double-blind, randomized, controlled, parallel-group, multi-site, clinical trial evaluating the efficacy and safety of Qutenza® in subjects with post-surgical neuropathic pain	Allocation: randomized intervention model: parallel assignment | masking: quadruple (participant, care provider, investigator, outcomes assessor) primary purpose: treatment
21	PNI	NCT05432973	Recruiting	11	Efficacy of percutaneous electrical nerve stimulation compared with passive assisted neurodynamics in brachial plexus neuropathy: a pilot randomized controlled trial	Allocation: randomized intervention model: parallel assignment |masking: triple (participant, investigator, outcomes assessor) | primary purpose: treatment
22	TN	NCT05217628	Recruiting	200	A Phase II/III, multicentre, 8-week run-in phase followed by a 12-week, prospective, parallel-group, double-blind, randomized withdrawal, placebo-controlled study, with a 52 week open label extension, to evaluate the efficacy and safety of daily 1.5 to 3.5 mg basimglurant in patients with pain associated with trigeminal neuralgia with suboptimal response to their current anti-pain therapy.	Allocation: randomized | intervention model: parallel assignment | masking: double (participant, investigator) primary purpose: treatment
23	TN	NCT05142228	Recruiting	40	Erenumab as a therapeutic approach for the management of trigeminal neuropathic pain (tnP)	Allocation: randomized intervention model: parallel assignment | masking: triple (participant, investigator, outcomes assessor) | primary purpose: treatment
24	TN	NCT04505280	Recruiting	40	Greater occipital nerve and cervical region injection in patients with trigeminal neuralgia	Allocation: randomized intervention model: parallel assignment masking: none (open label) primary purpose: treatment
25	CINP	NCT04468230	Recruiting	40	Phase 2 study of nicotine for the treatment of pain associated with chemotherapy-induced peripheral neuropathy	Allocation: N/A intervention model: single group assignment | masking: none (open label) primary purpose: supportive care
26	CINP	NCT05322889	Recruiting	35	Prevention of paclitaxel-induced neuropathic pain by telmisartan in patients with planned paclitaxel chemotherapy due to ovarian or breast cancer (PrevTel)	Allocation: N/A intervention model: single group assignment | masking: none (open label) primary purpose: prevention
27	GPNP	NCT04431778	Recruiting	64	Evaluation of the efficacy and tolerance of low doses of ethosuximide in the treatment of peripheral neuropathic pain	Allocation: randomized | intervention model: parallel assignment | masking: quadruple (participant, care provider, investigator, outcomes assessor) | primary purpose: treatment
28	GPNP	NCT04480164	Recruiting	76	A randomized double-blind ascending-dose placebo-controlled study of N-desmethylclobazam in patients with peripheral neuropathic pain	Allocation: randomized | intervention model: sequential assignment masking: double (participant, investigator) | primary purpose: treatment
29	GPNP	NCT04981925	Recruiting	300	Predictors of pain relief from mindfulness-based stress reduction in multiple forms of chronic pain patients	Allocation: N/A | intervention model: single group assignment | masking: none (open label) | primary purpose: basic science
30	SCI	NCT04379011	Active, not recruiting	24	Brivaracetam to reduce neuropathic pain in chronic SCI: a pilot clinical trial	Allocation: randomized | intervention model: parallel assignment | masking: quadruple (participant, care provider, investigator, outcomes assessor) | primary purpose: basic science
31	SCI	NCT04700033	Completed	23	Immersive virtual reality for chronic neuropathic pain after spinal cord injury: a feasibility trial	Allocation: randomized | intervention model: parallel assignment | masking: triple (participant, investigator, outcomes assessor) | primary purpose: treatment

PDN, Painful diabetic neuropathy; PHN, postherpetic neuralgia; SC, Sciatica; PNI, peripheral nerve injury; TN, Trigeminal neuralgia; CINP, Chemotherapy-induced Neuropathic Pain; GPNP, general peripheral neuropathic pain; SCI, Spinal cord injury neuropathic pain.

NCT03176472 is a phase II trial of ricolinostat (ACY-1215), a histone deacetylase 6 (HDAC6) inhibitor developed by Regenacy Pharmaceuticals LLC for treating PDN (Table 2, Line 2). Mechanical allodynia, cognitive impairment, and depressive-like behavior caused by spared nerve ligation (SNL) model were attenuated by continuous intraperitoneal injection of ricolinostat (ACY-1215). Moreover, ACY-1215 administration could suppress SNL-induced neuroinflammatory responses in the ipsilateral spinal dorsal horn, hippocampus and prefrontal cortex (Chen et al., 2022). It is worth noting that cytoplasmic HDAC6 regulates the acetylation of various non-histone proteins in healthy neurons (LoPresti, 2020). Hence, the side effects of ricolinostat (ACY-1215) should not be ignored.

NCT04146896 has completed a novel small molecule called NYX-2925 that modulates the N-methyl-D-aspartate receptor (NMDAR) (Table 2, Line 3). Blocking NMDAR could affect the central pain processing, thereby reducing chronic pain and other associated symptoms (Houck et al., 2019). NYX-2925 modulates NMDARs in a highly specific and selective manner to normalize NMDAR-dependent plasticity, which is a possible mechanism for the long-duration effects observed in these pain models. Notably, NYX-2925 is varying from ketamine (an NMDAR antagonist), rather than fully turning the receptor “on” (agonist) or “off” (antagonist). It is more likely to normalize NMDAR function, thus enhancing communication between neural cells and avoiding the issues associated with overactivation (pain sensitivity) or excessive inhibition (loss of protective reflexes) of NMDAR that have plagued NMDAR-targeted drug development historically. Since no drugs targeting NMDAR have been applied clinically and NMDAR plays an important role in NP, NYX-2925 is expected to be a new direction for NP treatment.

#### 4.1.2. Potential physical means

NCT05322746 is mainly concentrated on focal muscle vibration (FMV), a technique that applies a vibratory stimulus to a specific muscle or its tendon using a mechanical device (Table 2, Line 4). FMV is an innovative form of vibration with non-invasive intervention, which promotes neuroplasticity and durable motor recovery (Murillo et al., 2014). The vibration of a specific muscle can increase the motor-evoked potential, enhance the changes in corticospinal excitability, and produce involuntary contraction in the vibrated muscle (Chandrashekhar et al., 2021). FMV is expected to be a better approach than whole body vibration (WBV), which is effective for reducing PDN-associated pain over the two-and four-week intervals and has persistence in pain reduction beyond the day of treatment (Kessler et al., 2020). Chandrashekhar and colleagues’ pilot work demonstrated a significant improvement in average pain by wearable FMV applied to individuals with PDN (Chandrashekhar et al., 2021). The above preliminary results of wearable FMV are fascinating. Such type of FMV device is simple to wear and easy to use, which may change the management of PDN.

Other potential treatments for PDN include transcranial direct current stimulation (tDCS) (NCT05469074), repetitive transcranial magnetic stimulation (rTMS) (NCT 04833660), moxibustion (NCT05104047), LX9211 (NCT04455633, this drug will be explained below) (Table 2, Line 5–8) and so on. Drug therapy remains the main way to treat PDN, and physiotherapy is of great significance for PDN treatment. However, there is a lack of evidence on the use of combination therapies in combating PDN. The largest combination trial, COMBO-DN, compared the combination of pregabalin and duloxetine at moderate doses with high-dose monotherapy, and found neither approach to be statistically superior (Tesfaye et al., 2013).

### 4.2. Postherpetic neuralgia

PHN is defined as pain persisting for 3 months after the onset or healing from herpes zoster (HZ), a condition caused by Varizella-Zooster virus (VZV). The disease is caused by reactivation of a latent VZV infection in the sensory ganglia. Pain is the most prominent symptom in 90% of HZ patients (Zhang et al., 2022). The incidence of PHN continues to rise steadily, especially in older patients, possibly due to weakened immunity. PHN is considered as a chronic pain syndrome, which may be attributed to its persistence in a subset of patients over a long period of time (Pappagallo et al., 2000). The standard treatment is a combination of antivirals and pain medications. The commonly used painkillers include topical lidocaine patches, pregabalin, gabapentin and antidepressants (Cui et al., 2018). Although various treatments are available, patients, especially those with chronic PHN, are perpetually disappointed with the effectiveness of pain management and had to endure adverse drug reactions. Therefore, higher requirements for the treatment of PHN need to be put forward. The current trials working on PHN are summarized as follows.

#### 4.2.1. Potential drugs

In NCT04662281, a highly potent and selective adaptor-associated kinase 1 (AAK1) inhibitor, LX9211, is being tested (Table 2, Line 9). AAK1 belongs to the class of numb-associated family of protein kinases (NAKs), including BMP-2-inducible kinase (BIKE/BMP2K) and cyclin G-associated kinase (GAK). AAK1 is widely expressed in the brain, spinal cord, and dorsal root ganglia. LX-9211 demonstrated good efficacy in the STZ-induced diabetic PNP model and CCI model at oral doses of 0.3–1 mg/kg (Luo et al., 2022). The antinociceptive effects of AAK1 inhibitors were induced by the impaired endocytosis of the cell surface levels of μ2-containing GABAA channels. LX-9211 had completed phase I clinical trials and obtained a favorable safety and pharmacokinetics profile supportive of once-daily dosing. This compound received fast track designation by the FDA, and is currently in phase II trials for PDN (NCT04455633) and PHN (NCT04662281). More encouragingly, LX-9211 has significant and consistent benefits in the treatment of PDN, as reported by Lexicon pharmaceuticals Inc. at the 16th annual pain therapeutics summit.

NCT04664530 is a recruiting trial that works on esketamine, a well-known NMDA receptor inhibitor (Table 2, Line 10). Esketamine has both analgesic and antidepressant effects with few incidences of respiratory depression, delirium, hallucinations, nausea and vomiting (Riggs and Gould, 2021). Esketamine has an anti-depressant effect by increasing extracellular glutamate and mTORC1-dependent synapse formation (Shinohara et al., 2021). Anticonvulsants and antidepressants are clinically first-line drugs for the treatment of PHN, which are effective to treat PHN and alleviate patients’ depressive mood due to pain (Anosike et al., 2022). It should be taken into account that the long-term use of esketamine is addictive, limiting its application in treating chronic PHN. Esketamine is more effective against the acute-phase PHN (Riggs and Gould, 2021).

NCT04313335 is a prospective, randomized, open-label, endpoint-blinded study to investigate the preventive efficacy of the prophylactic use of duloxetine during acute herpes zoster (no complication, presents with vesicles within 72 h) on PHN in Beijing Tiantan Hospital (Table 2, Line 11). Duloxetine is a first-line drug for NP by inhibiting the reuptake of serotonin and norepinephrine (Marks et al., 2009). However, preventive intervention on PHN is still rare. A retrospective study revealed that the administration of gabapentin during the acute HZ period significantly decreased the incidence of PHN (Ghanavatian et al., 2019). Prophylactic medication is a novel choice and direction for relieving PHN.

#### 4.2.2. Potential physical means

Electroacupuncture (EA) is a traditional therapy for pain relief via stimulating related acupuncture points (Heo et al., 2021). NCT04560361 was conducted by Nanjing University of Chinese Medicine to explore the effect of EA on PHN (Table 2, Line 12). Another trial NCT03584061 is a pilot study to investigate autologous fat grafting on chronic PHN (Table 2, Line 13). A clinical observation study reported that autologous fat grafting might relieve chronic pain resulting from HZ (Sollie et al., 2021).

In recent years, considerable attention has been paid to the treatment of PHN. Except for those mentioned above, some clinical trials demonstrated that the new calcium channel blocker HSK16149 (NCT05140863) might have a better therapeutic effect (Table 2, Line 14). Efforts to prevent VZV infection at source in different populations will be essential to improve the current poor outcomes associated with HZ infection (Zhang et al., 2022). Prophylactic medication is the current direction for the treatment of PHN, including HZ vaccines for elder people (Lang and Aspinall, 2021). Nevertheless, more drugs warrant further investigations in the near future.

### 4.3. Sciatica

SC is clinically diagnosed based on the symptoms of radiating pain in one leg with or without associated neurological deficits. SC can lead to severe discomfort and functional limitation with a prevalence of approximately 2–5% (Younes et al., 2006). Patients with SC usually reported aching and a sharp leg pain radiating below the knee and into the foot and toes, or coexisting with low back pain. The pain was described as a sudden or slow onset and varied in severity (Ropper et al., 2015). Inflammation or compression of the lumbosacral nerve roots (L4-S1) forming the sciatic nerve is accounted for SC (Valat et al., 2010). Disk herniation resulting from age-related degenerative changes is the most common cause (Konstantinou et al., 2015). Most patients improved over time with conservative treatment, including exercise, manual therapy and pain management (Jensen et al., 2019). Surgery was performed on serious or progressive neurologic deficits such as motor weakness or bladder dysfunction (Deyo and Mirza, 2016). However, pain medications had uncertain benefit for SC and might have adverse effects (Jensen et al., 2019), while surgery methods had unsatisfactory locating and injection issues. Novel potential treatments on SC are being tested in several clinical trials.

#### 4.3.1. Potential drugs

In NCT03347929, a traditional non-selective NSAID, naproxen, is being tested, which has been approved for the treatment of inflammatory rheumatic conditions, osteoarthritis, primary dysmenorrhea, and musculoskeletal pain (Grøvle et al., 2022; Table 2, Line 15). However, the application of NSAIDs on SC remains a debate. Cochrane review of the existing trials found that NSAIDs were no more effective for pain reduction in SC patients compared with placebo or other drugs (Rasmussen-Barr et al., 2016). However, a survey indicated that 80% American physicians would recommend NSAIDs for the initial management of SC (Webster et al., 2005). The actual effect of NSAIDs on SC needs to be investigated further.

NCT05000658 is recruiting subjects for testing the efficacy of soluble dexamethasone in refractory SC (Table 2, Line 16). Dexamethasone is a synthetic corticosteroid with anti-inflammatory and immunosuppressive properties. National Institute for Health and Care Excellence (NICE) guidelines recommended the epidural injection of local anesthetic and steroid in the lumbar nerve root area for patients with acute and severe SC, where they would otherwise be considered for surgery (Jensen et al., 2019). The current trial is a phase III prospective randomized double-blind trial, which measures the efficacy of soluble dexamethasone by echo-guided infiltration through the sacro-coccygeal hiatus in intractable SC. This is an innovative operation, but its effectiveness may depend on the injection site.

In NCT05196503, the preventive effect of platelet-rich fibrin (PRF) on postsurgical NP after disk herniation surgery is being explored (Table 2, Line 17). PRF was isolated from venous blood without anticoagulant (Choukroun and Ghanaati, 2018). It is a natural fibrin matrix containing cytokines, platelet-derived growth factor (PDGF), bone morphogenetic protein (BMP), insulin-like growth factor-1 (IGF-1), transforming growth factor-β1 (TGF-β1), and vascular endothelial growth factor (VEGF) (Wu et al., 2016). PRF has increasingly been used in regenerative medicine and oral medicine due to its anti-inflammatory and analgesic properties. This effort may be a beneficial trial for preventing postsurgical NP.

#### 4.3.2. Potential physical means

NCT04875741 demonstrates the comparative effects of Neural Mobilization (NM) and Muscle Energy Technique (MET) on SC (Table 2, Line 18). MET was developed by Fred Mitchell. It is a type of physical therapy, and is classified as a common conservative treatment for SC and other pathologies of the spine (Santos et al., 2022). MET is a form of manual or ‘hands-on’ therapy used by osteopathic physicians, chiropractors, and physical therapists. In this treatment type, the muscles of a patient are contracted by pushing against resistance provided by the therapist. The therapist then assists the patient in stretching, strengthening and relaxing those muscles. The goal of MET is to help restore normal muscle and joint mobility (Franke et al., 2015). A meta-analysis indicated that MET was unlikely to improve lumbar dysfunction, but might help reduce the intensity of low back pain, with moderate efficacy in subacute SC (Franke et al., 2015). NM is a movement-based treatment modality used in treating pathologies of the nervous system, which includes both slider and tensioner maneuvers (Plaza-Manzano et al., 2020). The underlying mechanisms of neural mobilization interventions include the restoration of homeostasis in and around the nerves and attenuation of intraneural edema through intraneural fluid dispersion in the nerve root and axon (Boudier-Revéret et al., 2017). Neurodynamic treatment demonstrated a significant reduction in leg pain intensity and improved function after 4 weeks of intervention in patients with chronic SC (Ferreira et al., 2016). The current trial provides strong evidence for the effectiveness of these two methods on SC.

In NCT05129540, whether the foot orthoses can alleviate SC is being evaluated (Table 2, Line 19). This is based on the idea that chronic SC may be related to abnormal stresses applied to the musculoskeletal system during the gait cycle due to foot alterations (Attal and Bouhassira, 2021). However, the trial is not yet recruiting participants.

It is helpful for patients to understand the natural course of SC to relieve symptoms such as pain (Jensen et al., 2019). Treatment of SC is still dominated by physical therapy and supplemented by surgery, but the effectiveness of these medications remains uncertain. Therefore, SC patients may require more effective treatments, including new physiotherapy approaches, advanced surgical methods and novel drug therapies.

### 4.4. Peripheral nerve injury-related neuropathic pain

PNI-NP is a heterogeneous group of NP conditions caused by a peripheral nerve lesion, e.g., during surgery or trauma (Finnerup et al., 2021). There was a significant correlation between the risk of nerve damage during surgery and the risk of developing chronic NP (Haroutiunian et al., 2013), while the severity of injury and the type (transecting, stretching, crushing) of nerve damage were not significantly associated with the development of NP (Andersen et al., 2014). However, it remains unclear that most patients with nerve damage would develop pain while others did not. The percentage of those patients who experienced pain ranged from 67 to 95% (Teixeira et al., 2015). PNI-NP treatment is subjected to the standards of NP therapy and has special features. A follow-up with an experienced psychotherapist is strongly recommended (Mathews, 2014). Drug treatment, such as tricyclic antidepressants and calcium channel ligands (Teixeira et al., 2015), should be initiated early, which can be either aggressive or progressive. Surgical treatment is another option. Decompression, reconstruction, ablation and modulation are the four basic options for the surgical management of PNI-NP. The most optimal methods and means have been investigated by several researchers in order to better mitigate PNI.

#### 4.4.1. Potential drugs

NCT04967664 was performed to confirm the efficacy and safety of the repeated topical application of Qutenza (capsaicin 8% topical system) versus low-dose capsaicin control (capsaicin 0.04% topical system) in subjects with moderate-to-severe postsurgical NP (Table 2, Line 20). Capsaicin 8% patches are recommended as a second-line therapy for NP, which can be applied for 30–60 min and repeated every 3 months (Finnerup et al., 2021). The possible mechanism underlying this analgesic effect is that capsaicin binds to TRPV1, which is important for pain signal transduction, and both single and repeated application of high capsaicin doses can lead to the desensitization and dysfunction of TRPV1. The most commonly reported adverse events are application site reactions, including dryness, erythema, oedema, pain, papules and pruritus. The analgesic effect of 8% capsaicin patch was better than that of 0.04% capsaicin patch in PHN patients, but the adverse event rates were higher (Backonja et al., 2008). However, it remains unclear how the capsaicin 8% topical system works with PNI-NP.

#### 4.4.2. Potential physical means

In NCT05432973, the efficacy of transcutaneous electrical nerve stimulation (TENS) versus passive-assisted neurodynamics for pain relief in brachial plexus neuropathy is compared (Table 2, Line 21). TENS intervention was defined as pulsed electrical currents generated by a ‘standard TENS device’ administered across the intact surface of the skin using surface electrodes at the site of pain or over nerve bundles proximal (or near) to the site of pain, with the intention of stimulating peripheral nerves to alleviate pain (Johnson, 2021). TENS is a neuromodulation therapy, which has been widely used for symptomatic relief of pain. Physiological evidence suggests that TENS could inhibit the activity and excitability of central nociceptive transmission neurons (Johnson et al., 2022). A meta-TENS study provides moderate-certainty evidence that pain intensity is lower during or immediately after TENS compared with placebo, and without any serious adverse events (Johnson et al., 2022). Hence, TENS is a potential alternative for the treatment of PNI-NP.

The first-line therapy for PNI-NP is etiology treatment. Repair of peripheral nerve injury should be considered, such as reconnecting the damaged nerve through surgery, as well as medication or physical therapy to promote nerve repair. A variety of drugs have been used to relieve PNI-NP, while the nerve damage is hard to repair. The above trial might provide us with more possibility of providing adjuvant treatment on PNI-NP.

### 4.5. Trigeminal neuralgia

TN is the most common form of craniofacial NP disorder characterized by spontaneous and elicited paroxysms of electric shock–like or stabbing pain affecting one side of the face. Triggered paroxysmal pain is specific to TN, and is reported in 91–99% of NP patients (Di Stefano et al., 2018), indicating that this feature may be pathognomonic of TN. Intracranial vascular compression of the trigeminal nerve root is the main cause of TN (Cruccu et al., 2020). Carbamazepine and oxcarbazepine are recommended as first-line therapies for TN (Cruccu et al., 2008). Surgery is performed only if the standard doses of medications are not sufficient to control the symptoms or the side effects prevent their continuous use (Cruccu et al., 2020). Nonetheless, the first-line drugs have serious side effects, including dizziness, diplopia, ataxia and elevated transaminase levels, resulting in 23% of patients discontinuing medications (Cruccu et al., 2014). Thus, it is necessary to develop new treatment modalities for TN.

#### 4.5.1. Potential drugs

NCT05217628 is a phase II/III trial to test the efficacy and safety of basimglurant (Table 2, Line 22). Basimglurant is a potent, selective, and safe metabotropic glutamate subtype 5 (mGlu5)-negative allosteric modulator with good oral bioavailability and long half-life supportive of once-daily administration in humans (Quiroz et al., 2016). mGluR5 is vital for the release of neurotransmitters and regulating the postsynaptic response of neurotransmitters in the central and peripheral nervous systems. mGlu5 at synaptic and extrasynaptic locations can enhance the activity of NMDA receptors with increasing NMDA currents. Inhibiting the downstream effects of mGlu5 receptor could down-regulate the function of NMDA (Tebano et al., 2005). Basimglurant might achieve positive results for the treatment of TN, except for its previous application on major depression.

In NCT05142228, the TN pain-related outcomes after injection of aimovig under the skin are being evaluated (Table 2, Line 23). Aimovig (erenumab) is the first antibody therapy targeting the calcitonin gene-related peptide (CGRP) receptor with FDA approval for migraine prevention with well tolerated and good safety profile (Wang et al., 2021). CGRP plays a pivotal role in the formation and maintenance of NP (Xie et al., 2017; Wang et al., 2021). This trial is an expanded application of aimovig (erenumab) on TN.

#### 4.5.2. Potential physical means

NCT04505280 was conducted to investigate the efficacy of greater occipital nerve block and cervical injections with lidocaine (Table 2, Line 24). The pharmacological block of the greater occipital nerve has been proven to be effective in numerous headache and facial pain syndromes (Hoffmann et al., 2021). Nociceptive signals induced by stimulation of peripheral trigeminal afferents are transmitted along the trigeminal nerve passing the trigeminal ganglion and enter the brainstem into the trigeminocervical complex (TCC) (Goadsby et al., 2017). Nociceptive information originating in the occipital region of the head is transmitted through the occipital nerve into the TCC (Angus-Leppan et al., 1997). Previous animal studies suggest that neuronal signals originating from trigeminal and occipital neurons converge in the TCC, and within the TCC, around two-thirds of neurons receive afferent input from trigeminal and occipital neurons (Angus-Leppan et al., 1997). An occipital nerve block may inhibit neurons in TCC and relieve TN.

### 4.6. Chemotherapy-induced neuropathic pain

CINP in cancer patients is usually caused by neurotoxic chemotherapeutic agents that lead to peripheral neuropathies (Staff et al., 2017). CINP is one of the painful polyneuropathies, and is a severe adverse reaction that affects up to 80% of patients during cancer treatment (Sisignano et al., 2016). To date, there are no FDA-approved drugs for preventing or treating CINP, with emerging debate on its underlying mechanisms and therapeutic targets (Bae et al., 2021).

NCT04468230 was performed to assess the efficacy of short-term nicotine transdermal patch administration for the treatment of CINP in cancer stable or remission patients (Table 2, Line 25). Nicotine, the main stimulant in cigarette smoke, was found to have antinociceptive effects in animal and clinical studies (Rowley et al., 2008). A nicotine patch allows less variability in plasma nicotine with a slow steady level delivered through the skin over time. A systematic review and meta-analysis indicated that nicotine patches had an opioid-sparing effect in non-smokers who underwent surgery after general anesthesia, but did not significantly reduce the pain scores (Mishriky and Habib, 2014). Landim et al. found that nicotine patch was effective at controlling pain following third molar surgery (Landim et al., 2020). Nevetheless, the current clinical trial is a meaningful attempt under the condition of local anesthesia, rather than that of general anesthesia.

In NCT05322889, the effectiveness of telmisartan, an angiotensin AT1 receptor blocker, in CINP patients is being tested (Table 2, Line 26). Treatment with telmisartan in mice could lead to a strong reduction of CINP. Interestingly, its analgesic effect was associated with the inhibition of cytochrome P450 oxidase 2 J (CYP2J) rather than angiotensin AT1 receptor (Sisignano et al., 2016). Telmisartan has been on the market for a long time and has a good risk profile, low occurrence of side effects, and is generally well tolerated in patients. Hence, telmisartan may be a promising pharmacological treatment option for CINP patients.

### 4.7. Others

Numerous clinical trials are not focused on a particular type of PNP, thus, we classified them as GPNP.

NCT04431778 is a phase II trial that assesses the efficacy and tolerance of low ethosuximide (ETX) doses in GPNP patients (Table 2, Line 27). ETX is a low voltage-activated (LVA) calcium channel blocker, which completely removes painful neuropathic symptoms induced by sciatic and spinal nerve ligation in mice (Dogrul et al., 2003; Kerckhove et al., 2019). However, a proof-of-concept, randomized, double-blind and controlled trial (NCT02100046) showed that although ETX had an analgesic effect on NP *in vivo*, it was not effective for NP patients at a certain dose (30 ml (1,500 mg) per day) (Kerckhove et al., 2018). The current trial aims at testing the effect of low ETX doses on NP patients, which can further strengthen the clinical application of LVA calcium channel blockers.

In NCT04480164, the safety and efficacy of N-desmethylclobazam (NDMC) in GPNP patients are being evaluated (Table 2, Line 28). NDMC is the main metabolite of clobazam (CBZ), and may act as a GABAA receptor inhibitor (Ralvenius et al., 2016). CBZ is a family of benzodiazepine (BDZ), which has a sedative, anti-convulsive, analgesic and antianxiety effect. Previous studies demonstrated that classical BDZ activated α1-subtype GABAA receptors (Ralvenius et al., 2015) and α2-subtype GABAA receptors (Paul et al., 2014), leading to severe side effects (e.g., sedation, addiction and motor impairment). Moreover, the data from transgenic mice also supported that the beneficial effects of antipain could be induced by selective allosteric regulation of GABAA receptors without any cognitive and calming effects (Knabl et al., 2008). NMDC has a high selectivity profile with an obvious preference for GABAA α2-subtypes receptors rather than α1 receptors, which is mainly responsible for its sedative effects (Ralvenius et al., 2016). Since NDMC naturally occurs as the metabolite of CBZ, together with the safety profile known from its parent drug CBZ, it is very unlikely that NDMC has unexpected adverse reactions. Thus, GABA is not a common target for NP treatment, and its possible interaction with NDMC is worthy of further investigation.

In NCT04981925, the predictors of pain relief for mindfulness-based stress reduction (MBSR) are identified (Table 2, Line 29). MBSR is one of the mindfulness-based interventions, and individuals primarily have a non-judgmental attitude toward all experiences (Majeed et al., 2018). MBSR exhibited beneficial effects on the physical and mental health of patients with chronic pain compared to cognitive behavioral therapy (CBT) (Veehof et al., 2011). MBSR treatment had a greater improvement at 26 weeks in low back pain compared with usual care (NCT01467843) (Cherkin et al., 2016). Additionally, MBSR is effective against pain reduction, as well as improvements in physical function, mood and sleep disturbance in patients with chronic pain (Burns et al., 2022). MBSR is a potential therapeutic modality for chronic NP without any side effects. The current trial (NCT04981925) is helpful for the identification of potential molecular/physical targets in NP patients.

## 5. Central neuropathic pain

### 5.1. Spinal cord injury-related neuropathic pain

SCI-NP is defined as pain associated with a lesion or disease of the somatosensory nervous system, and remains difficult to treat (Finnerup et al., 2016). SCI-NP is typically experienced at or below the zone of injury, and is classified as sharp, burning or electric (Bryce et al., 2012). Principles of SCI-NP treatments include self-management, psychological interventions, pharmacological treatment, neuromodulation, and personalized medicine (Attal, 2021). SCI-NP is minimally responsive to the existing pharmacological interventions (Boldt et al., 2014), which may have serious adverse events. The intractable nature of SCI-NP is a strong impetus to explore alternative therapeutic approaches.

In NCT04379011, whether brivaracetam can reduce SCI-NP is being explored (Table 2, Line 30). Brivaracetam (an antiepileptic drug) is a synaptic vesicle protein 2 (SVP2) ligand, which displays similar selectivity, but higher affinity than its parent compound levetiracetam (Kenda et al., 2004). Tsymbalyuk et al. reported that brivaracetam significantly reduced microglial activation, TNF expression, and leukocyte infiltration into the dorsal horn, in conjunction with reduced NP behaviors in a murine sciatic nerve injury model (Tsymbalyuk et al., 2019). In real-world clinical practice, brivaracetam is sometimes used to relieve TN, either as monotherapy or in combination (Pergolizzi et al., 2022). Since brivaracetam is a ligand of SVP2, and the latter is a potential target of NP (Kenda et al., 2004), it can serve as a mechanism-based pharmacological intervention for SCI-NP.

NCT04700033 showed that immersive interactive virtual walking could reduce NP, leading to a significant decline in depressive symptomatology among patients with SCI (Trost et al., 2022; Table 2, Line 31). Immersive virtual reality (IVR) provides a completely immersive experience for participants and gives users the feeling of being in a virtual world. However, the mechanism of action is still unclear. The study’s sample size was small, and participants were not randomly assigned to study conditions, thus introducing a potential bias. IVR acts as a new type of wearable device, and may be a promising treatment for SCI-NP.

SCI-NP has lifelong impacts on health and well-being. Therefore, improving the management of this complicated pain condition is an important goal. The translation of knowledge between basic and clinical research, as well as the clinical management of NP after SCI can be realized by uncovering the reliable and valid clinical NP phenotypes (e.g., pain type, pain symptoms, sensory function and dysfunction, and psychosocial factors), translatable biomarkers, and therapeutic targets (Widerstrom-Noga, 2017).

## 6. Discussion

In this review, we briefly summarize the existing clinical trials on the treatment of NP to enrich the knowledge of potential novel therapeutic strategies, including drug targets, new implications of conventional medicine and physical means. NP treatment is mainly dominated by drug therapy, and physical means have become increasingly popular. These potential strategies offer new hope for NP treatment and encourage us to uncover the mechanisms of NP heterogeneity.

Drug therapy remains the dominant mode of NP therapy. With the development of basic science and technology, there have been increasing numbers of novel molecular targets and molecular compounds of NP being discovered and synthesized, respectively. The AAK1 inhibitor LX9211, which is effective against NP relief in rats, has great potential for PHN treatment (NCT04662281). More importantly, uncovering the mechanisms of NP helps the clinicians to explore the new implications of conventional drugs for pain relief. For example, NDMC is the first drug used to treat NP by targeting GABAARs (NCT04480164). Erenumab might treat TN like its application on migraine since they share the same CRGP pathway (NCT05142228). Optimizing the dosage and duration of conventional drugs might also work well. The current trials have compared the effects of different concentrations of capsaicin patches on various types of NP (NCT04967664), or prophylactic use of duloxetine to reduce PHN (NCT04313335). With a deeper understanding of the mechanisms of NP, we believe that there is great potential for the application of new medicines in the clinical setting.

The inevitable problems with medication are adverse events and possible analgesic effects on different types of NP. Adverse reactions of gabapentin and pregabalin include dizziness, drowsiness, confusion and fatigue, and they are ineffective for SC. Local injection of botulinum toxin A has a beneficial effect on PNI, but a detrimental effect on other types of NP. A large-scale study found that the combination of nortriptyline with morphine at moderate doses was more effective than monotherapy at higher doses for the treatment of PNP (Gilron et al., 2015). The combination of different drugs may reduce potential side effects without diminishing the analgesic effect. Several clinical trials are under way on the combinations of different drugs, which may be a future direction to eliminate or reduce the side effects to a bearable level.

In addition to drug therapies, physical means are getting popular. Patients who enhance their strength and endurance through physical therapy for general health often report a reduction in pain compared with before treatment (Mathews, 2014). Neuromodulation therapy is one of the successful means for NP by promoting neuroplasticity (Knotkova et al., 2021), which includes deep brain and motor cortex stimulation, peripheral nerve stimulation, and the non-invasive treatments of repetitive transcranial magnetic stimulation, transcranial direct current stimulation and TENS. A meta-analysis revealed the analgesic effect of noninvasive brain stimulation (NIBS) on patients with NP, and recommended its clinical application (Gao et al., 2022). Another systematic review and meta-analysis proved the analgesic effect of rTMS on NP and stimulus sites. The combination of stimulus frequency and site as well as the location of pain are critical modulators of the rTMS effect (Jiang et al., 2022). However, the placebo effect of neuromodulation could not be neglected (Knotkova et al., 2021).

With the advancement of technology, several novel physical approaches are shown to be effective in preventing NP, including VR and Sana Pain Reliever (Sana PR). VR could improve patients’ mental state and reduce patients’ anxiety (Trost et al., 2022). The movement of SCI-NP patients could be simulated with the aid of VR. Sana PR is another device to provide Audio Visual Stimulation (AVS), which enhances performance stability and symptom management. These novel devices might help clinicians to have multiple choices in treating NP patients individually.

The current study has some limitations. Firstly, we only searched clinical trials on ClinicalTrials.gov and might miss out potential trials in other databases. Secondly, we did not consider trials completed over 5 years as the inclusion criteria. Thirdly, the current search was done on December 8, 2022 to avoid frequent updates of the database. Practitioner could update the current knowledge on ClinicalTrials.gov with the search term “Neuropathic Pain” in the “Condition or disease” dialog. Those who are interested in a specific pain sub-type or treatment type could apply an advanced search with the search terms of a specific pain sub-type in “Other terms” dialog or choose the “Study type” (Interventional or Observational) below. Fourthly, less attention has been paid to multimodal pain management programs, drug combination therapy, dietary therapy and psychological intervention. Several of them proved their effectiveness for chronic pain. For example, dietary supplementation of gingerols–and shogaols-enriched ginger root extract could attenuate pain-associated behaviors (Shen et al., 2022). Omega-3 fatty acids may be of great benefit in the management of NP patients (Ko et al., 2010). It is worth noting that the combined approaches have become increasingly important in treating complex NP. We paid less attention to those combination treatment, as most of the trials were related to gabapentin or pregabalin, the first-line therapies for patients with NP. Additionally, only a few trials (51/914, 5.6%) reported on the combined therapy on NP. In the future, it is important to consider conducting basic studies to uncover the potential mechanisms of combined therapy, as well as mechanism-based clinical studies. Fifthly, the reasons for retired or terminated trials might provide valuable information for further guidance on NP treatment. However, this information could not be found directly through ClinicalTrials.gov. Trial design, small sample size, failure to achieve the endpoint of outcome, and potential toxic effects in both animal and human experiments could be the reasons behind a retired or terminated trial. Thus, is imperative to consider the potential biases in the study design when interpreting the results. Finally, CNP was seldom discussed and some rare types of NP were not covered, such as occipital neuralgia, pudendal neuralgia and NP in Parkinson’s disease. Despite these limitations, the current review provides researchers and clinicians with new hopes for the treatment of NP and encourages us to invent more therapeutic approaches.

## 7. Conclusion

NP remains difficult to treat and represents a huge unmet medical need. NP treatment is mainly dominated by drug therapy, and physical means have become increasingly popular. It should be highlighted that novel drug targets, new implications of conventional medicine, and novel physical means are the promising strategies for NP treatment. Nevertheless, more attention needs to be paid to the challenges of translating research findings into clinical practice.

## Author contributions

ZD and JZ collected and analyzed the data. ZD, JZ, and XH reviewed and confirmed the data analysis. ZD, JZ, WY, and XG wrote the manuscript. WY and XG designed the project and revised the manuscript. All authors contributed to the article and approved the submitted version.

## Funding

This work was supported by Technological Innovation 2030-Major Projects of “Brain Science and Brain-like Research” (2022ZD0206200) to XG, Shanghai Rising-Star Program (21QC1400300) to XG, the promoting project of Renji Hospital (RJTJ23-RC-013) to XG, the Medical Engineering Cross Research Foundation of Shanghai Jiao Tong University (YG2022QN035) to XH, National Natural Science Foundation of China (Nos. 81701092, 81971223, and 32030043) to XG and WY, and Shanghai Engineering Research Center of Peri-operative Organ Support and Function Preservation (20DZ2254200) to WY.

## Conflict of interest

The authors declare that the research was conducted in the absence of any commercial or financial relationships that could be construed as a potential conflict of interest.

## Publisher’s note

All claims expressed in this article are solely those of the authors and do not necessarily represent those of their affiliated organizations, or those of the publisher, the editors and the reviewers. Any product that may be evaluated in this article, or claim that may be made by its manufacturer, is not guaranteed or endorsed by the publisher.
